# Inhibition of Let7c MicroRNA Is Neuroprotective in a Rat Intracerebral Hemorrhage Model

**DOI:** 10.1371/journal.pone.0097946

**Published:** 2014-06-24

**Authors:** Jeong-Min Kim, Soon-Tae Lee, Kon Chu, Keun-Hwa Jung, Jin Hee Kim, Jung-Suk Yu, Soyun Kim, So Hee Kim, Dong-Kyu Park, Jangsup Moon, Jaejun Ban, Manho Kim, Sang Kun Lee, Jae-Kyu Roh

**Affiliations:** 1 Laboratory for Neurotherapeutics, Biomedical Research Institute, Seoul National University Hospital, Seoul, South Korea; 2 Department of Neurology, Chung-Ang University Medical Center, Seoul, South Korea; 3 Department of Neurology, Seoul National University Hospital, College of Medicine, Seoul National University, Seoul, South Korea; Duke University, United States of America

## Abstract

Intracerebral hemorrhage (ICH) is a devastating neurological disease with a grave prognosis. We evaluated microRNA (miRNA) expression after ICH and evaluated Let7c as a therapeutic target. We harvested hemorrhagic brain 24 hours after collagenase induced ICH in the rat. Microarray analysis was performed to compare the miRNAs expression pattern between hemorrhagic hemisphere and contralateral hemisphere. An in vitro thrombin toxicity model and blood injection ICH model were also used to evaluate miRNA expression. We selected miRNA for the therapeutic target study after reviewing target gene databases and their expression. The antagonistic sequence of the selected miRNA (antagomir) was used to evaluate its therapeutic potential in the in vitro thrombin toxicity and in vivo ICH models. Among 1,088 miRNAs analyzed, let7c was induced in the thrombin and ICH models. Let7c antagomir treatment increased cell survival in the in vitro thrombin injury model and improved neurological function at 4 weeks after ICH. Let7c antagomir decreased perihematoma edema, apoptotic cell death and inflammation around hematoma. Let7c antagomir also induced insulin like growth factor receptor 1 (IGF1R) protein and phosphorylated serine threonine kinase after ICH. This study shows a distinct miRNA expression pattern after ICH. The let7c antagomir reduced cell death and edema and enhanced neurological recovery at least in part by activating the IGF1R pro-survival pathway. This suggests blocking let7c might be a potential therapeutic target in ICH.

## Introduction

Intracerebral hemorrhage (ICH) is one of the most devastating stroke subtypes with grave prognosis and has the highest mortality [1], [2]. Despite outstanding progress in ischemic stroke treatment technology in the past ten years, little progress has been achieved in ICH treatment. Several novel treatment strategies including factor VIIa and neuroprotective agents have shown discouraging outcomes, and current therapeutic options for ICH are still limited to supportive management such as blood pressure control or treatment of complications [2], [3]. Development of novel treatment strategies based on the distinct pathogenic mechanism of ICH is warranted for successful clinical application.

MicroRNA (miRNA) is a short sequence non-coding RNA with 20–25 base pairs which regulates gene expression in the post-transcription step by base-pairing with the target messenger RNA of the 3′ untranslated region [4]. Its expression level is dynamic in the development stage of humans and in diseases, and several studies have confirmed the therapeutic potential of modulating miRNA expression [4]–[6]. Recently we studied the miRNA expression pattern in a human Alzheimer's disease (AD) brain sample and Tg2576 AD transgenic mouse brain, and found that the level of miR-206, which regulates brain derived neurotrophic factor (BDNF), was markedly increased in AD mice [7]. The inhibition of miR-206 by intranasal antagonistic sequence administration increased brain BDNF and improved memory function [7]. We also showed that multiple miRNAs were increased in a mouse stroke model and proved its modulation had neuroprotective potential in the in vitro oxygen glucose deprivation condition [5]. Regarding ICH, there has been one study reporting miRNA expression patterns in animal models, but no study has investigated the therapeutic potential of miRNA modulation [8]. Considering previous messenger RNA expression analysis in ICH animal models and human brains reporting down-regulation of cell survival pathways and increased inflammatory gene expression, it is expected that miRNA directed gene modulation could be a feasible therapeutic approach in ICH [9.10].

In this study we tried to evaluate the miRNA expression pattern in a rat collagenase induced ICH model to understand ICH specific pathophysiology and to discover therapeutic targets by miRNA modulation. Using miRNA microarray and quantitative real-time polymerase chain reaction (qRT-PCR), we selected candidate miRNA with potential therapeutic effect. Whether its inhibition by counter sequenced miRNA, namely antagomir (AM), has a neuroprotective effect was studied using the in vitro thrombin toxicity model and in vivo ICH models.

## Materials and Methods

### Intracerebral Hemorrhage Model

This study was approved by the Institutional Animal Care and Use Committee of Seoul National University Hospital, Korea. Male Sprague-Dawley rats (Daehan Bio, Seoul, Korea) weighing 200 to 220 g were used in the experiment. Experimental ICH was induced by stereotactic administration of bacterial collagenase type VII, as described previously (injection point: 3.0 mm left lateral to the midline, 0.2 mm posterior to bregma, 6.0 mm in depth below the skull) [11], [12]. We also constructed two additional ICH models to confirm miRNA expression pattern of collagenase induced ICH, by injection of 300 uL blood at the same coordinate as the collagenase infusion site as described previously [13], or by injection of the equal volume of normal saline to produce mass effect (n = 6 per each model). All surgical procedures were performed under anesthesia to minimize suffering. The animals were raised with a 12-h light/dark cycle in a specific pathogen free facility and ad libitum access to food and water. The animals were deeply anesthetized by intraperitoneal injection of 1% ketamine (30 mg/kg) and xylazine hydrochloride (4 mg/kg) before sacrifice.

### MicroRNA Microarray and Quantitative Real Time Polymerase Chain Reaction

24 hours after ICH induction, four rats were sacrificed and their brains were harvested. Total miRNA was extracted from each hemisphere with a miRNeasy Mini Kit (Qiagen, CA, USA), and the concentration and quality of each RNA sample was determined using the Agilent 2100 Bioanalyzer (Agilent Technologies, CA, USA). The miRNAs from each hemisphere were labeled and hybridized by using the GenoExplorer microRNA array labeling kit and GenoExplorer microRNA chips including 1,088 miRNAs (GenoSensor Corporation, AZ, USA). The hybridized microRNA chips were scanned and analyzed using an Axon GenePix 4000B scanner and GenePix Pro software (Molecular Devices, CA, USA). The heat map pattern of miRNA expression was visualized according to Z-score, which is calculated by (raw individual value of the miRNA expression-mean of the miRNA expressions)/(standard deviation of the miRNA expression), and analyzed by paired t-test.

Selected miRNA from the microarray was validated by using the GenoExplorer miRNA qRT-PCR kit (GenoSensor Corporation, AZ, USA) and miRNA specific primer (GenoSensor Corporation, AZ, USA). Using the comparative threshold cycle (Ct), relative expression was calculated from the formula: relative expression  = 2^−ΔCt^, and was normalized with the expression of control RNU6B (GenoSensor Corporation, AZ, USA) from each sample. The seed sequences of miRNAs in this study are as follows: let7c, 16-UGAGGUAGUAGGUUGUAUGGUU-37; miR-24-2, 25-GUGCCUACUGAGCUGAAA CAGU-46; miR-142-5p, 16-CAUAAAGUAGAAAGCACUACU-36.

### Targeted Gene Prediction and AntagomiR Design

We reviewed four major miRNA target prediction sites to find potential target genes of elevated miRNAs; these prediction sites were TargetScan (www.targetscan.org), Pictar (http://.pictar.mdc-berlin.de/), microRNA.org (www.microrna.org), and miRecords (http://mirecords.biolead.org/). The miRNA which was up-regulated in the ICH hemisphere and was predicted to regulate genes of potential therapeutic effect was selected and its antagonistic sequences of single stranded RNAs with cholesterol residue at 3′ ending and phosphorothioate backbone was constructed to neutralize the targeted miRNAs (Bioneer, Daejon, Korea).

### *In Vitro* Thrombin Toxicity Model and Therapeutic Potential Study

To simulate neuronal injury after ICH, an in vitro thrombin toxicity model was constructed by applying thrombin (Sigma, NY, USA) to rat pheochromocytoma cell (PC12) line (ATCC, VA, USA). The culture media consisted of RPMI 1640 (Gibco BRL, NY, USA), 10% horse serum (Sigma, NY, USA), 5% fresh bovine serum (Sigma, NY, USA), penicillin G (Sigma, NY, USA) 100 u/ml, and streptomycin (Sigma, NY, USA) 100 ug/ml which were placed in a chamber with 5% CO2 and 100% humidity. Thrombin was applied at constant concentration and the cell viability assay was measured by the WST-1 assay kit (Roche, Basel, Switzerland), according to the manufacturer's instructions. After applying various concentrations of thrombin, we found that 200 U/ml thrombin administration resulted in 50% cell death and 500 U/ml thrombin administration resulted in 90% cell death after 24 hours. The therapeutic potential of AM was studied from the in vitro thrombin injury model. The cells were transfected by selected AM with Lipofectamine 2000 (Invitrogen, CA, USA).

### *In vivo* Therapeutic Application Study

In a previous experiment we showed that intranasal delivery of AM is a convenient and efficient way of modulating miRNA expression in a neurological disease model [7]. Intranasal 5 nmol AM in 24 ul of 0.1% diethylpyrocarbonate-treated distilled water was applied immediately after ICH induction by pipette in 4 ul drops on each nostril alternatively [7]. The control group received the same amount of vehicle. Five rats from each group were anesthetized and the brains were removed 72 hours after ICH induction to obtain the wet weight by measuring immediately on an electronic balance and dry weight after drying for 24 hours in a gravity oven. Water contents are expressed as the percentage of wet weights and calculated by the following equation: [(wet weight-dry weight)/(wet weight) X 100 (%)] [11], [12].

For immunochemistry, seven rats from each group were anesthetized and perfusion of the heart was maintained with 50 mL cold saline and 50 ml of 4% paraformaldehyde, in 0.1 mol/l of phosphate buffered saline. The brain sections were stained with antibodies against myeloperoxidase (MPO, 1∶200, DAKO, MD, USA) and Ox-42 (1∶500, Chemicon, CA, USA) as described previously [11], [12]. Apoptotic cell death was evaluated by terminal deoxynucleotidyl transferase dUTP nick end labeling (TUNEL) staining which detects DNA fragmentation during apoptosis [11], [12]. Positively stained cell numbers were quantitatively analyzed in the perihematomal regions of three axial sections through the center of the hemorrhagic lesion. Total cell counts in three sections were then converted to cell densities in cortex and basal ganglia separately.

Modified limb placing test every week until the fifth week was performed by two investigators unaware of the experimental groups (n = 5 per group) to evaluate functional improvement as described previously with slight modification [11], [12].

### Western Blot Analysis

Four anesthetized animals from each group were decapitated after perfusion of the heart with 10 mL cold saline and homogenates of each brain were serially processed for western blotting. For in vitro study we transfected 70 nmol/L of the candidate mature double stranded miRNA with neuroprotective potential or scrambled miRNA control (Bioneer, Daejon, Korea) to the PC12 cell line using lipofectamine 2000 (Invitrogen, CA, USA) and evaluated the potential target protein after 24 hours, as described previously [5]. Western blotting was carried out using antibodies against insulin like growth factor 1 receptor (IGF1R; Santa Cruz, CA, USA), IGF2R (Santa Cruz, CA, USA), high mobility group AT-hook 2 (HMGA2; Cell Signaling Technology, MA, USA), IGF2 binding protein 1 (IGF2BP1; Abcam Incorporated, MA, USA), glyceraldehydes 3-phosphate dehydrogenase (GAPDH; Cell Signaling Technology, MA, USA), serine threonine protein kinase (Akt; Santa Cruz, CA, USA), and phosphorylated Akt (p-Akt; Santa Cruz, CA, USA). The relative optical densities of each protein band by normalization with GAPDH were calculated using Image-J program (National Institutes of Health, MD, USA).

### Statistical Analysis

Continuous values were expressed as mean value ± standard deviation. The Mann-Whitney U test was used to compare values between the two groups. Analysis of variance for repeated measures followed by the Bonferroni method for post-hoc inter-group comparison was performed to evaluate recovery of neurological function. Two tailed p-value less than 0.05 was considered as statistically significant.

## Results

### MicroRNA Expression Profile in ICH Model and in vitro Thrombin Injury Model

**Figure 1:**
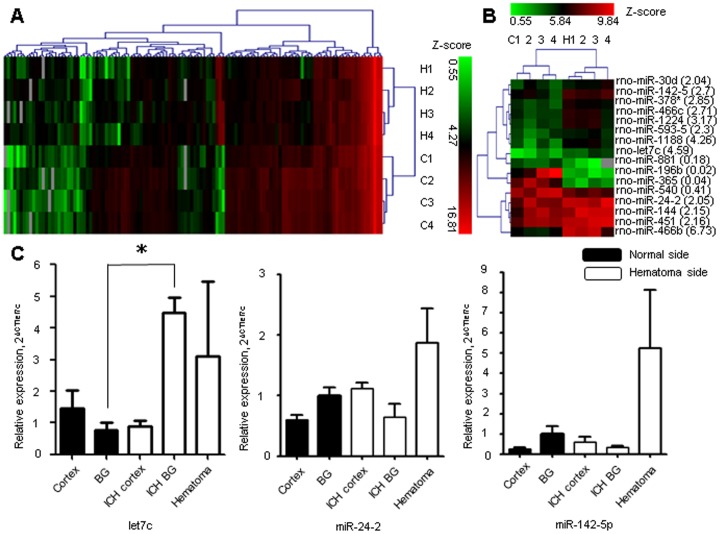
The microRNA expression after intracerebral hemorrhage. The heat map shows the microRNA (miRNA) expression pattern from the microarray between the left hemorrhagic hemisphere and right non-hemorrhagic hemisphere, with statistically significant difference (p<0.05) by paired t-test (A), and the difference of more than two folds by paired t-test (p<0.05) (B). The level of let7c was increased in the basal ganglia in the region specific manner by quantitative real time polymerase chain reaction study (C). *P<0.05, n = 4–5 per group, H, hemorrhagic hemisphere, C, contralateral hemisphere, BG, basal ganglia, ICH, intracerebral hemorrhage.

The microarray result showed several miRNAs were up-regulated in the hemorrhagic hemisphere compared to the opposite hemisphere (figure 1A, B). Those miRNAs which were significantly elevated in hemorrhagic hemisphere and have broadly conserved sequence throughout species were selected for further evaluation. Since both human hypertensive ICH and animal model of collagenase induced ICH are primarily located in the basal ganglia, the miRNA expression pattern was studied in the cortex and basal ganglia separately. We found that let7c was increased robustly in the basal ganglia 24 hours after ICH induction (figure 1C).

**Figure 2:**
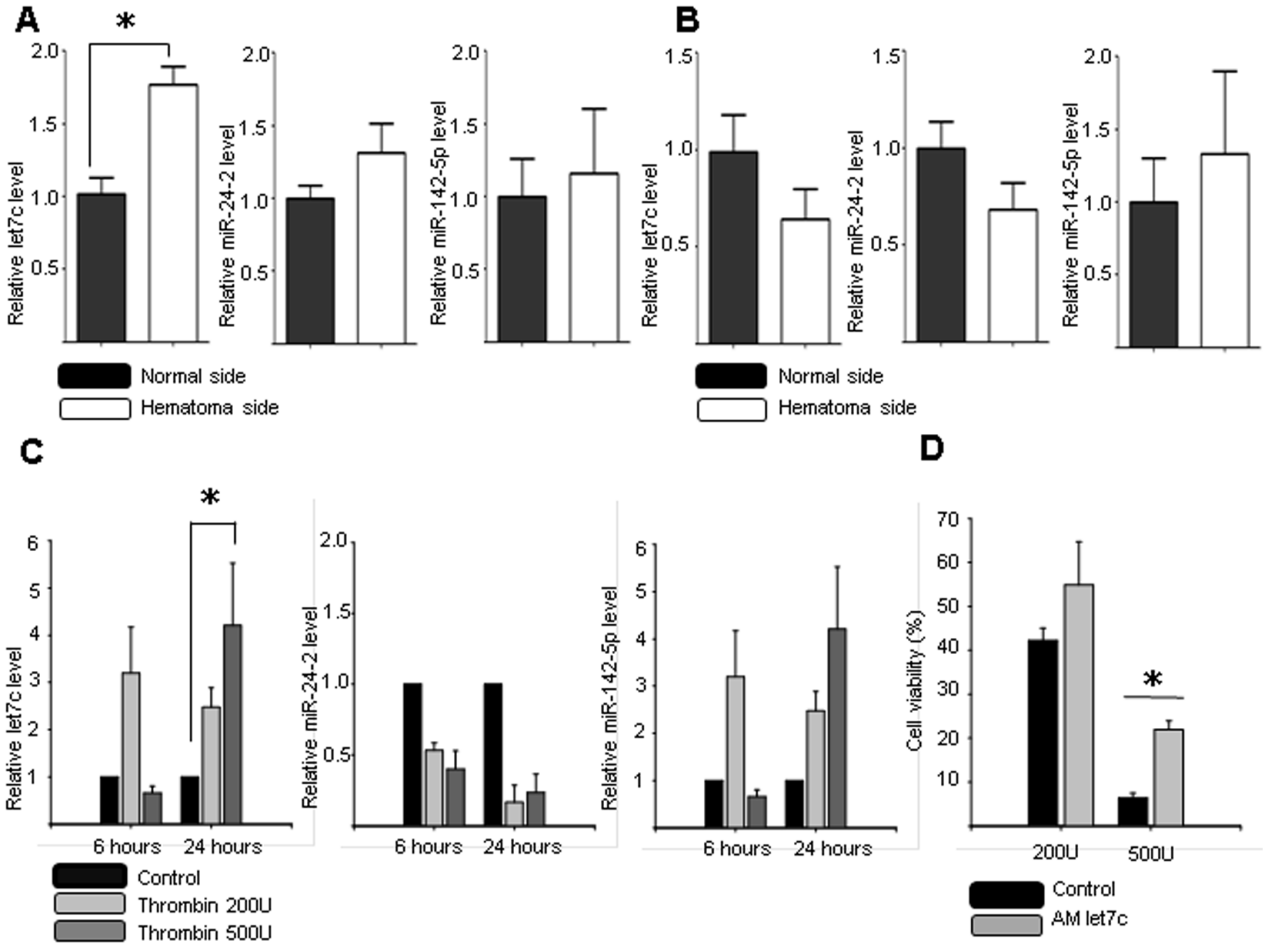
The microRNA expression in blood injection model, saline injection model, and in vitro thrombin injury model. The level of let7c increased after blood injection on the same coordinate as collagenase (p = 0.0159) (A), but not after normal saline injection (p = 0.0937) (B). The in vitro thrombin injury model showed that let7c increased 24 hours after the 500 U thrombin injury (C). The let7c antagomir (AM) increased cell survival from the thrombin injury (6.38±1.09% vs. 21.89±2.01%, p = 0.037) (D). *P<0.05, n = 5–6 per group.

Next the miRNA expression level was evaluated from two additional ICH models and in vitro thrombin injury model. The level of let7c increased significantly in blood injection model, but not in saline injected model (figure 2A, B). The level of let7c increased significantly 24 hours after thrombin injury (figure 2C). We selected let7c to validate its expression pattern and the protective effect of the inhibitory sequence because it is a broadly conserved miRNA throughout species and consistently elevated in various ICH models. The application of AM let7c for 24 hours increased cell viability at the 500 U thrombin injury (6.38±1.09% vs. 21.89±2.01%, p = 0.037; figure 2D).

### In vivo AntagomiR Treatment Effect

**Figure 3:**
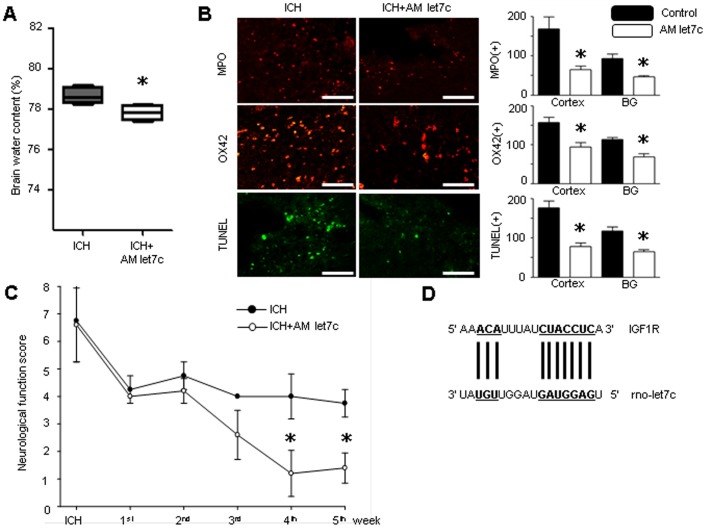
The neuroprotective effect of let7c modulation in the intracerebral hemorrhage model. The antagomir (AM) let7c delivery via the intranasal route attenuated brain water content 72 hours after intracerebral hemorrhage (ICH) (78.7±0.4% vs. 77.4±0.4%, p = 0.029) (A). The numbers of perihematoma neutrophils labeled by myeloperoxidase (MPO) and Cyanine 3 (red) in cortex (158±32 vs. 94±29, p = 0.009) and basal ganglia (113±12 vs. 69±20, p = 0.002) were decreased by the AM let7c treatment 72 hours after ICH (B, Bar = 100 µm). The number of activated microglia labeled by OX42 and Cyanine 3 was also decreased by AM let7c (cortex 168±75 vs. 65±22, p = 0.009; basal ganglia: 93±28 vs. 46±11, p = 0.002) (B, Bar = 100 µm). Apoptotic cell death marked by terminal deoxynucleotidyl transferase dUTP nick end labeling (TUNEL) staining and fluorescein isothiocyanate staining (green) was decreased by AM let7c in cortex (177±41 vs. 78±23, p = 0.004) and basal ganglia (118±25 vs. 65±11, p = 0.002) (B, Bar = 100 µm).Neurological function tests showed that AM let7c facilitated neurological recovery at four weeks after ICH (C). The representative binding site of rno-let7c on rat insulin like growth factor 1 receptor messenger RNA is illustrated (D). *P<0.05, n = 7 per group, IGF1R, insulin-like growth factor 1 receptor.

The immediate intranasal application of AM let7c was not associated with any noticeable adverse event throughout experiment, and abrogated the brain water content (78.7±0.4% vs. 77.4±0.4%, p = 0.029) (figure 3A). Inflammatory neutrophil infiltration, marked by MPO (cortex: 158±32 vs. 94±29, p = 0.009; basal ganglia: 113±12 vs. 69±20, p = 0.002), and activated microglia marked by OX42 (cortex 168±75 vs. 65±22, p = 0.009; basal ganglia: 93±28 vs. 46±11, p = 0.002) at 72 hours after ICH were decreased in cortex and basal ganglia by AM let7c (Figure 3B). The apoptotic cell death measured by TUNEL staining 72 hours after ICH (cortex: 177±41 vs. 78±23, p = 0.004; basal ganglia: 118±25 vs. 65±11, p = 0.002) were also decreased by AM let7c.The AM let7c treatment showed noticeable functional improvement 4 weeks after ICH (Figure 3C). By the in silico target prediction programs, let7c was predicted to control IGF1R expression, which is known to be part of a pro-survival pathway (Figure 3D).

### Let7c Pathway Study

**Figure 4:**
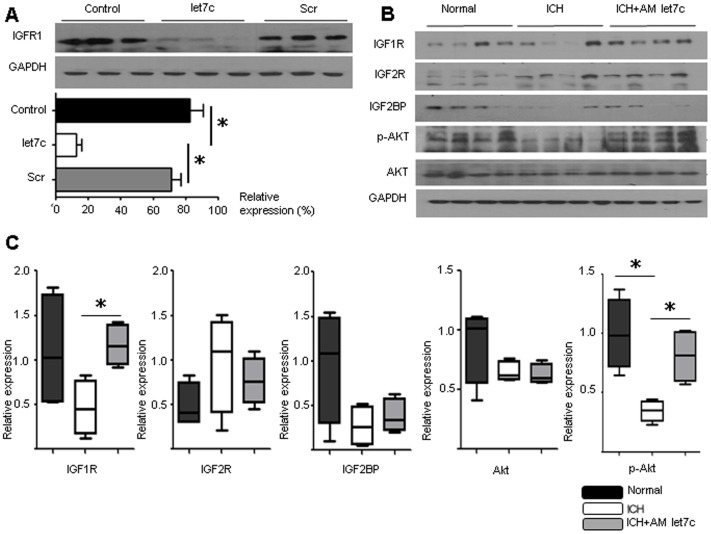
Increased Insulin-like Growth Factor 1 Receptor Protein and Pro-survival Signal by let7c Modulation. The transfection of let7c in the PC12 cell line significantly decreased insulin like growth factor 1 receptor (IGF1R) protein (A). The western blot analysis of potential let7c targets, including IGF1R, IGF2R, and IGF2 mRNA binding protein 1 (IGF2BP1) from the hemorrhagic hemisphere was compared with the normal control hemisphere and the non-treated hemorrhagic group (B). The quantitative analysis showed that IGF1R protein was decreased by intracerebral hemorrhage (ICH), and was significantly restored by antagomir (AM) let7c treatment (C). The phosphorylated serine threonine kinase (p-Akt) was markedly increased after AM let7c (C). *P<0.05, n = 4 per group, Scr, scrambled, GAPDH, glyceraldehydes 3-phosphate dehydrogenase.

The transfection of let7c in PC12 cells decreased IGF1R protein expression significantly compared to either the negative control or transfection of scrambled miRNA sequence, which suggests that let7c determines IGF1R expression (Figure 4A). The in vivo expression level of IGF1R protein was decreased at 72 hours after ICH, but the AM let7c treated group showed restored IGF1R expression (Figure 4B, C). The levels of IGF2R, IGF2BP1, and total Akt did not change significantly with AM let7c treatment. However, p-Akt expression, which is an IGF1 pathway prosurvival signaling molecule, was restored by AM let7c (Figure 4B, C).

## Discussion

This study shows the miRNA expression pattern after ICH induction, and the results indicate that up-regulated miRNAs could be a potential therapeutic target in ICH. When let7c was abrogated by its antagonist application, neuronal survival was increased in the in vitro thrombin toxicity model and functional improvement was facilitated after ICH, with reduced apoptotic cell death. The IGF1R expression was influenced by let7c modulation, and the activated cell survival pathway after IGF1R signaling is proposed as a plausible therapeutic mechanism of AM let7c.

This study shows the characteristic miRNA expression profile in the ICH model with region-specific elevation. The expression pattern of miRNA had been previously reported from three different brain hemorrhage models produced by intraventricular autologous blood, lysed blood and thrombin injection [8]. They showed that miR-298 and miR-245 increased in the blood injected ICH model, and that there was a miR-107, miR-200b and miR-331-5p increment in the thrombin injection model [8]. The miRNA expression pattern of our study is different from the results of the previous study because the researchers harvested the hippocampus for miRNA extraction, although hippocampus is not the principally injured site in either the collagenase induced ICH model or human ICH patient. We evaluated regional differences of miRNA expression after ICH and found that let 7c was up-regulated more than four folds in the basal ganglia compared to the contralateral side. Considering that the basal ganglia are one of the most frequent ICH locations in the clinical field, it is rational to suspect a pathophysiologic role of let7c and to study its modulation effect in ICH. Blood degradation product such as thrombin might be important in let7c induction, because let7c expression was also increased in blood injection model, but not in saline injection model. There has been a recent report studying the circulating blood miRNA level in ICH patients, showing that there is a let7f increment in patients with early hematoma enlargement [14].

The role of let7 has been widely studied in cancer and in stem cell biology, and its potential targets include multiple anti-apoptotic and cell proliferation pathways [15]. The insulin signaling network has been known as a cell survival pathway, which is mediated by insulin receptors of two types, insulin receptor and IGF1R [16], [17]. Their activation phosphorylates Akt and the mammalian target of rapamycin proteins, which induces pro-survival protein synthesis and regulates apoptosis related proteins [16]. The neuroprotective effect of AM let7c is probably mediated by IGF1R up-regulation with decreased apoptotic cell death around the hematoma of 50% compared to controls. Recently the neuroprotective effect of let7f antagomir which is one base pair different from let7c was studied in the cerebral infarction animal model [18]. It showed robust expression of let7f from the ischemic cortex, and the antagonistic sequence of let7f significantly increased IGF1 expression level and decreased the infarct volume [18]. Another study showed the neuroprotective effect of IGF1 and erythropoietin combination treatment via intranasal delivery in the middle cerebral artery occlusion model [19]. Considering several previous studies disclosing the neuroprotective effect of IGF1R activation, it is conceivable to suspect that the IGF1R signal can be a promising therapeutic target in ICH.

The possible anti-inflammatory effect of AM let7c treatment is another interesting finding. There exist several lines of evidence suggesting IGF1 signaling relates to inflammation [20], [21]. One study showed that IGF-1 infusion delayed atherosclerotic lesion progression in ApoE-deficient mice by reducing vascular inflammation and inflammatory cytokines [21]. Previous study of let7f in a cerebral infarction model showed let7f is primarily localized in the microglia by in situ hybridization combined with immunohistochemistry, which is related to reduced IGF signaling [18]. It is also probable that there exist other targets of let7c which control the inflammatory cascade after neuronal injury. Recent study found that let7 can function as signaling molecule of Toll-like receptor 7, and contributes to neurodegeneration by activating the innate immune receptor [22]. On the other hand, decreased apoptotic cell death by activation of the IGF1R pathway may have indirectly reduced reactive inflammatory cell recruitment around the hematoma. Future studies focusing on inflammatory cytokine production and immune cell regulation by let7c modulation will help to increase understanding of its role in inflammation modulation.

Intranasal delivery has been suggested as a convenient and effective transmission modality for central nervous system acting medications [7], [23]. The proposed route of administration is along the olfactory and trigeminal neural pathways from the nasal mucosa to the brain and the drug enters the perivascular spaces from which it is rapidly dispersed throughout the brain [24]. Not only drugs, but cells, viral vectors and peptides have been successfully delivered to the brain via intranasal administration [23], [25]. It can bypass first-pass metabolism and allow the direct delivery of the drug to the cerebral spinal fluid [24]. Our group administered antagomiR of miR-206 in the Alzheimer's disease transgenic mouse model, and showed a cognitive enhancing effect by increasing the BDNF [7]. Another group showed enhanced delivery efficiency of IGF1 via the intranasal route compared to the intravenous or intraperitoneal routes in a cerebral infarction model [19]. This is another study showing effective intranasal delivery of a miRNA antagonistic sequence to modulate brain pathology.

There are several weak points of this study. First of all, thrombin was applied on the PC12 cell line to imitate an in vitro ICH condition, but it is evident that more extensive mechanisms are related to post-hemorrhagic neuronal damage in the real clinical situation.^2^ It is impossible to simulate perihematomal inflammation or tissue hypoperfusion by an in vitro thrombin injury model. Considering that the PC12 cell line is from pheochromocytoma tissue, the miRNA expression level and protective effect of let7c derived from in vitro thrombin injury may be different from the brain tissue response after ICH. However, the neuroprotective effect of AM let7c in the in vitro thrombin injury model was validated in the vivo ICH model, in which apoptotic cell death was reduced by promoting IGF1R signaling. Only the intranasal delivery of AM let7c was studied in this study, although there is a chance that other administration routes such as intracerebral injection could be more efficient. Future studies regarding dose response, time window, as well as the route of administration will help to strengthen the feasibility of miRNA modulation strategy in ICH.

In conclusion, we demonstrated a distinct miRNA expression pattern after ICH and its modulation can have therapeutic potential. The let7c antagomir reduced cell death and inflammation, and enhanced neurological recovery by activating the IGF1R pro-survival pathway. This suggests blocking let7c might be a potential therapeutic target in ICH.

## Supporting Information

Checklist S1 **ARRIVE Checklist.**
(DOC)
