# Correction: Inhibition of Let7c MicroRNA Is Neuroprotective in a Rat Intracerebral Hemorrhage Model

**DOI:** 10.1371/journal.pone.0104465

**Published:** 2014-07-29

**Authors:** The PLOS ONE Staff

The Y-axes for the three graphs in Figure 1C are incorrectly labeled. Please see the complete, correct Figure 1 here.

**Figure 1:**
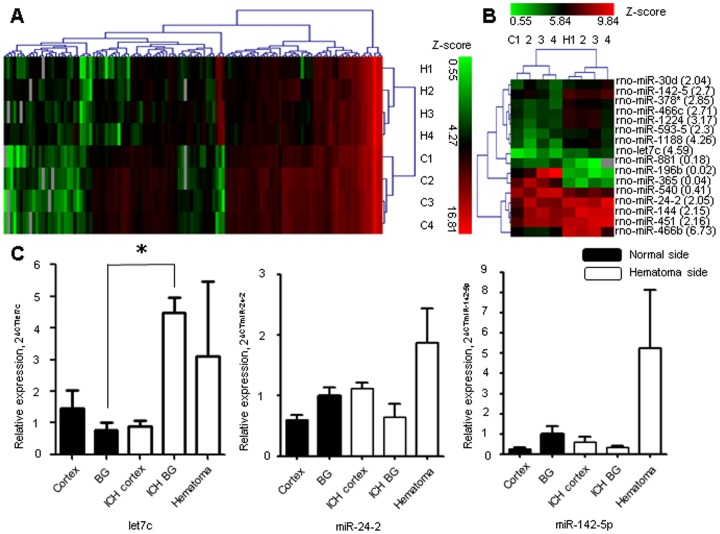
The microRNA expression after intracerebral hemorrhage. The heat map shows the microRNA (miRNA) expression pattern from the microarray between the left hemorrhagic hemisphere and right non-hemorrhagic hemisphere, with statistically significant difference (p<0.05) by paired t-test (A), and the difference of more than two folds by paired t-test (p<0.05) (B). The level of let7c was increased in the basal ganglia in the region specific manner by quantitative real time polymerase chain reaction study (C). *P<0.05, n  =  4–5 per group, H, hemorrhagic hemisphere, C, contralateral hemisphere, BG, basal ganglia, ICH, intracerebral hemorrhage.
